# Hydroxymethylglutaryl-CoA reductase inhibition with simvastatin in Acute lung injury to Reduce Pulmonary dysfunction (HARP-2) trial: study protocol for a randomized controlled trial

**DOI:** 10.1186/1745-6215-13-170

**Published:** 2012-09-17

**Authors:** Daniel F McAuley, John G Laffey, Cecilia M O’Kane, Mark Cross, Gavin D Perkins, Lynn Murphy, Christine McNally, Grainne Crealey, Michael Stevenson

**Affiliations:** 1 Centre for Infection and Immunity, Queen's University of Belfast, Belfast, BT9 7BL, UK; 2 Regional Intensive Care Unit, Royal Victoria Hospital, Belfast, BT12 6BA, UK; 3 Clinical Research Support Centre, Royal Victoria Hospital, Belfast, BT12 6BA, UK; 4 Department of Anaesthesia, Clinical Sciences Institute, National University of Ireland, Galway, Ireland; 5 Warwick Medical School Clinical Trials Unit, University of Warwick, Warwick, CV4 7AL, UK

**Keywords:** Simvastatin, Acute lung injury, Acute respiratory distress syndrome

## Abstract

**Background:**

Acute lung injury (ALI) is a common devastating clinical syndrome characterized by life-threatening respiratory failure requiring mechanical ventilation and multiple organ failure. There are *in vitro*, animal studies and pre-clinical data suggesting that statins may be beneficial in ALI. The Hydroxymethylglutaryl-CoA reductase inhibition with simvastatin in Acute lung injury to Reduce Pulmonary dysfunction (HARP-2) trial is a multicenter, prospective, randomized, allocation concealed, double-blind, placebo-controlled clinical trial which aims to test the hypothesis that treatment with simvastatin will improve clinical outcomes in patients with ALI.

**Methods/Design:**

Patients fulfilling the American-European Consensus Conference Definition of ALI will be randomized in a 1:1 ratio to receive enteral simvastatin 80 mg or placebo once daily for a maximum of 28 days. Allocation to randomized groups will be stratified with respect to hospital of recruitment and vasopressor requirement. Data will be recorded by participating ICUs until hospital discharge, and surviving patients will be followed up by post at 3, 6 and 12 months post randomization. The primary outcome is number of ventilator-free days to day 28. Secondary outcomes are: change in oxygenation index and sequential organ failure assessment score up to day 28, number of non pulmonary organ failure free days to day 28, critical care unit mortality; hospital mortality; 28 day post randomization mortality and 12 month post randomization mortality; health related quality of life at discharge, 3, 6 and 12 months post randomization; length of critical care unit and hospital stay; health service use up to 12 months post-randomization; and safety. A total of 540 patients will be recruited from approximately 35 ICUs in the UK and Ireland. An economic evaluation will be conducted alongside the trial. Plasma and urine samples will be taken up to day 28 to investigate potential mechanisms by which simvastatin might act to improve clinical outcomes.

**Trial registration:**

Current Controlled Trials ISRCTN88244364.

## Background

Acute lung injury (ALI) is a common devastating clinical syndrome characterized by life-threatening respiratory failure requiring mechanical ventilation and multiple organ failure. ALI is defined by the American-European Consensus Conference [1] as the acute onset of hypoxia (PaO2:FiO2 ratio of <40 kPa) and bilateral infiltrates on a chest radiograph in the absence of cardiogenic causes of pulmonary edema. ALI occurs in response to a variety of insults. It affects all age groups; has a mortality of approximately 30% to 40% [2-5] and causes a long-term reduction in quality of life for survivors [6]. ALI has significant resource implications, prolonging ICU and hospital stay, and requiring rehabilitation in the community with many survivors unable to return to work 12 months after hospital discharge [7,8].

### Rationale for statins in the treatment of ALI

Hydroxymethylglutaryl coenzyme A (CoA) reductase inhibition with statins is a potential novel therapeutic strategy to treat ALI. Statins have been shown to modify a number of the underlying mechanisms that mediate this process both *in vitro* and *in vivo* in animal models of ALI [9]. In an observational study in patients with ALI, after adjusting for potential confounding factors, patients receiving statins had a lower probability of death with an odds ratio 0.27, although this failed to reach statistical significance [4]. In healthy volunteers, pre-treatment with simvastatin for four days results in attenuation of both pulmonary and systemic inflammatory responses in a model of ALI induced by lipopolysaccharide (LPS) inhalation [10]. In this human model, statins reduced pulmonary neutrophilia, pro-inflammatory cytokines and proteases as well as systemic C-reactive protein (CRP).

A single center, randomized, double-blind, placebo-controlled study, Hydroxymethylglutaryl-CoA reductase inhibition with simvastatin in Acute lung injury to Reduce Pulmonary dysfunction (HARP), investigated simvastatin (80 mg for up to 14 days) in 60 patients with ALI [11]. This study provided proof of concept data of a beneficial effect with significant improvements in non-pulmonary organ dysfunction, as measured by the Sequential Organ Failure Assessment (SOFA) score as well as a trend to improvements in pulmonary dysfunction, as measured by the oxygenation index (OI), respiratory system compliance and lung injury score in the simvastatin-treated group. Importantly, simvastatin 80 mg was well tolerated with no increase in adverse events. In addition, pulmonary IL-8 and systemic CRP were lower in the simvastatin-treated group. The study was not designed or powered to show an effect of simvastatin on clinical outcomes. The findings from these studies helped inform the design of two large clinical trials. In the US, the National Heart, Lung, and Blood Institute Acute Respiratory Distress Syndrome (ARDS) network is currently conducting a trial of rosuvastatin for infection-related ALI (SAILS study; NCT00979121). The aim of the HARP-2 trial is to test the hypothesis that treatment with enteral simvastatin 80 mg would improve clinical outcomes in patients with ALI irrespective of etiology. A second objective is to determine the effects of simvastatin treatment on biological mechanisms important in the development of ALI and assess whether the response to simvastatin is determined by genetic polymorphisms.

## Methods/Design

The Belfast Health and Social Care Trust (BHSCT) is the sponsor for the UK sites and the National University of Ireland (NUI) Galway is the sponsor for the Irish sites. The trial will be conducted in accordance with the ethical principles that have their origin in the Declaration of Helsinki. The protocol is approved by the Office for Research Ethics Committees, Northern Ireland for UK sites (10/NIR02/36) and by the institutional Research Ethic Committee for each site in Ireland. The trial is registered on the International Standard Randomized Controlled Trial Registry (ISRCTN88244364) and with the European Union Drug Regulating Authorities Clinical Trials database (2010-020763-20). The study is funded by the Efficacy and Mechanism Evaluation (EME) program (http://www.eme.ac.uk). This study is also funded in Ireland by the Health Research Board. The HSC R&D, Public Health Agency in Northern Ireland and the Intensive Care Society of Ireland have also provided additional funding. The trial is being coordinated by the Clinical Research Support Centre (CRSC) as the Clinical Trials Unit (CTU) (http://www.crsc.hscni.net). The trial will comply with the principles of good clinical practice (GCP) and will be carried out in accordance with applicable legislation and the standard operating procedures of the CRSC. The trial will be reported in line with the Consolidated Standards of Reporting Trials (CONSORT) 2010 guidelines [12].

### Outcome measures

The primary outcome measure is ventilator free days (VFDs). VFDs will be defined as the number of calendar days from the time of initiating unassisted breathing to day 28 after randomization, assuming survival for at least two consecutive calendar days after initiating unassisted breathing and continued unassisted breathing to day 28. If a patient returns to assisted breathing and subsequently achieves unassisted breathing prior to day 28, VFDs are counted from the end of the last period of assisted breathing. If a patient was receiving assisted breathing at day 27 or dies prior to day 28, VFDs will be zero [13]. Unassisted breathing is defined as being extubated with supplemental oxygen or room air; or open T-tube breathing; or tracheostomy mask breathing; or continuous positive airway pressure (CPAP) ≤5 cm H_2_0 without pressure support. Sites are advised that usual CPAP or (BIPAP) Bilevel positive airway pressure solely for sleep-disordered breathing management is not defined as assisted breathing. Patients receiving pressure support via non-invasive ventilation for any other reason will be defined as receiving assisted ventilation. Patients transferred to another hospital or other health care facility will be followed to day 28 to assess this endpoint.

There are a number of secondary outcomes for this clinical trial which include clinical outcomes, safety, health economic evaluation and biological mechanisms.

The secondary clinical outcomes are change in OI from baseline to day 3, 7, 14 and 28; change in SOFA score from baseline to day 3, 7, 14 and 28; non pulmonary organ failure free days (defined as the number of days in the first 28 days after randomization that the patient received no cardiovascular, renal, liver or neurological support as defined by the Critical Care Minimum Dataset [14]); all cause mortality 28 days post randomization; mortality at (first) discharge from critical care; mortality at (first) discharge from hospital and mortality at 12 months post randomization.

### Safety

The frequency with which the following events occur will be reported: (1) creatine kinase (CK) >10 times the upper limit of normal; (2) alanine transaminase (ALT)/aspartate aminotransferase (AST) >8 times the upper limit of normal; (3) need for renal replacement therapy in patients with CK elevated >10 fold; and (4) serious adverse events (SAEs) and occurrence of suspected unexpected serious adverse reactions (SUSARs). Safety monitoring (CK and liver transaminases) is undertaken at days 3, 7, 14, 21 and 28.

### Health economic evaluation

Health related quality of life (HRQoL) will be measured using the EuroQol 5 dimension questionnaire (EQ-5D) [15]. Data will be collected at hospital discharge and 3, 6 and 12 months post randomization. Resource utilization (critical care and hospital length of stay and health service contact) will be recorded at 6 and 12 months post randomization.

### Biological mechanisms

A translational study of biological markers of inflammation and lung injury to provide insight into the mechanism of action of simvastatin in ALI will be undertaken. Blood and urine will be taken on days 0, 3, 7, 14 and 28 while patients continue to receive the study drug. Plasma from 20 ml of heparinized blood along with aliquots of urine will be stored at −70°C until analysis. We will measure neutrophil activation, proinflammatory cytokines and adhesion molecule expression and nuclear factor kappa B (NFkB) activation. Furthermore, we will measure cell-specific indices of activation and injury to the alveolar epithelium and endothelium, as well as the lung extracellular matrix degradation. Blood will also be collected for genetic testing to assess whether response to simvastatin is determined by genetic polymorphisms.

It is recognized that it may not be possible to collect plasma and urine samples from all patients and in the event that samples are not collected, this will not be recorded as a protocol violation.

### Eligibility criteria

Patients will be eligible for the trial if they fulfill the following criteria:

1. Receiving invasive mechanical ventilation and

2. ALI as defined by the American-European Consensus Conference criteria [1] of acute onset of:

a) Hypoxic respiratory failure (PaO2/FiO2 ≤40 kPa from two blood gases >1 hour apart).

b) Bilateral infiltrates on chest X-ray consistent with pulmonary edema. Infiltrates considered ‘consistent with pulmonary edema’ include any patchy or diffuse infiltrates not fully explained by mass, atelectasis, or effusion or opacities known to be chronic (>28 days).

c) No clinical evidence of left atrial hypertension or if measured, a pulmonary arterial occlusion pressure (PAOP) less than or equal to 18 mmHg.

All ALI criteria (a to c) must occur within the same 24 hour period. The time of onset of ALI is when the last ALI criterion is met.

Patients fulfilling any of the criteria below will be excluded from the trial:

1. Age <16 years

2. More than 48 hours from the onset of ALI

3. Patient is known to be pregnant

4. CK >10 times the upper limit of the normal range

5. Transaminases >8 times the upper limit of the normal range

6. Patients currently receiving ongoing and sustained treatment with any of the following: itraconazole, ketoconazole, HIV protease inhibitors, nefazodone, cyclosporine, amiodarone, verapamil or diltiazem.

7. Patients with severe renal impairment (estimated creatinine clearance less than 30 ml/minute) not receiving renal replacement therapy

8. Severe liver disease (Child's Pugh score >12)

9. Current or recent treatment (within two weeks) with statins

10. Physician decision that a statin is required for proven indication

11. Contraindication to enteral drug administration, for example, patients with mechanical bowel obstruction. Patients with high gastric aspirates due to an ileus are not excluded.

12. Domiciliary mechanical ventilation except for CPAP/BIPAP used for sleep-disordered breathing.

13. Known participation in other investigational medicinal product (IMP) trials within 30 days.

14. Consent declined

15. Treatment withdrawal imminent within 24 hours

16. Non-English speaking patients or those who do not adequately understand verbal or written information unless an interpreter is available.

### Power and sample size estimate

The mean (standard deviation; SD) VFDs in 432 patients with ALI was 12.7 (10.6) days [16]. The SD (10.6) for VFDs in ALI used for the sample size calculations is similar to the SD for VFDs that has been consistently reported in other large multi-center clinical trials [17-19]. There are no prospective trials in patients with ALI to predict the treatment effect size of simvastatin to improve VFDs. In a recent retrospective study, statin usage in patients with ALI was associated with a 31% increase in VFDs [20]. Our observational data showed a 37% relative improvement in mortality in patients who received a statin [4]. In our proof of concept study, OI and SOFA scores improved by 50% to 66%, respectively, in the simvastatin-treated group [11]. Pre-treatment with simvastatin decreased a range of pulmonary inflammatory mediators induced by LPS in healthy volunteers by between 34% to 65% [10]. On the basis of these data, a conservative treatment effect of 20% has been estimated for this study.

**Figure 1:**
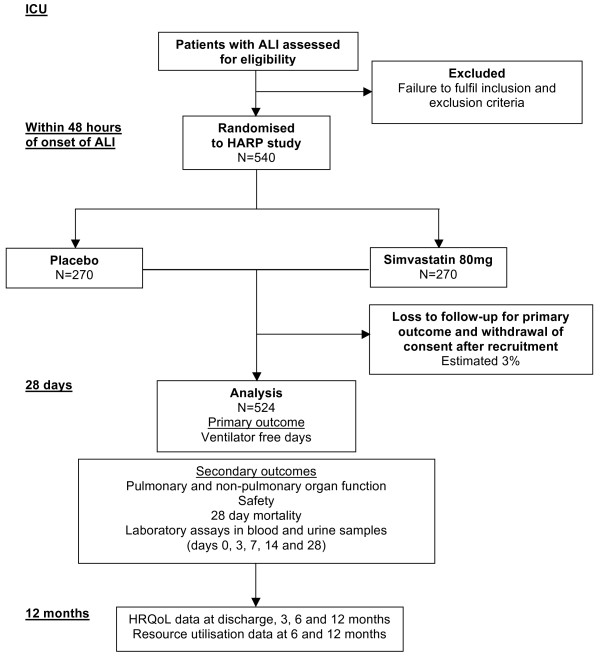
HARP-2 trial flow diagram.

A sample size of 524 subjects (262 in each group) will have 80% power at a two-tailed significance level of 0.05 to detect a 20% difference in VFDs. To estimate loss after recruitment, previous data from the PAC-Man trial were used where 2.4% of recruited patients or their relatives subsequently withdrew their consent, or were randomized in error [21]. Thus, estimating a dropout rate of 3% the study will require a total of 540 patients with 270 in each group (Figure 1).

## Trial conduct

### Consent

Informed consent will be obtained prior to conducting any trial specific procedures.

Critically ill sedated patients do not have the capacity to give consent; therefore, consistent with requirements of the EU clinical trial directive, we will obtain written informed consent/assent from a representative in keeping with regulatory requirements before randomization. All surviving patients will be informed about the trial at the earliest opportunity after regaining competence and consent to continue in the trial will be sought. The consent from the representative will remain valid until a decision on consent to continue is obtained from the patient.

### Randomization and study drug supply

Patient drug packs will be prepared by Victoria Pharmaceuticals (Belfast, UK). Simvastatin 40 mg or identical placebo tablets will be packaged in a white opaque plastic container which will be sealed with a tamper-evident seal and labelled in compliance with applicable regulatory requirements. Each container will contain 70 tablets of the study drug for the treatment of one patient for 28 days (plus 7 days overage). All trial drugs will be packaged identically and identified only by the unique trial identifier.

Patients will be randomized in a 1:1 ratio using an automated centralized 24-hour telephone or web-based randomization service (Centre for Healthcare Randomised Trials, University of Aberdeen, UK). Randomization will be stratified by site and by vasopressor requirement (defined as any inotropic requirement except dopamine <6 mcg/kg/minute). The randomization service will allocate a unique trial identifier to each patient in accordance with the computer-generated study randomization schedule. The randomization service will confirm randomization details by email to the CTU and the study site. A confirmation email will be sent to the hospital pharmacy. The clinician will complete a trial prescription form detailing the unique trial identifier assigned to the patient. The hospital pharmacy will dispense the drug pack labelled with the corresponding unique trial identifier for the patient.

### Study drug administration

Patients will be randomized to receive once daily simvastatin 80 mg (as two 40 mg tablets) or two identical placebo tablets administered enterally via a feeding tube or orally for up to 28 days. The first dose of the study drug will be administered as soon as possible, ideally within four hours of randomization and subsequent doses will be given each morning starting on the following calendar day. If for any reason a dose is not administered at the intended time, it will be administered subsequently but not more than 12 hours after the intended time of administration.

### Post randomization withdrawals and exclusions

Patients may withdraw or be withdrawn by their representative from the trial or the trial treatment at any time without prejudice. Data recorded up to the point of withdrawal will be included in the trial analysis, unless consent to use their data has also been withdrawn. If a subject withdraws, they will be followed-up wherever possible and data collected as per protocol until the end of the trial. The only exception to this is where the subject also explicitly withdraws consent for follow-up.

### Study drug termination criteria

The study drug will be discontinued if any one of the following are met, prior to the maximum treatment period (28 days from randomization):

1. CK >10 times the upper limit of normal (ULN)

2. ALT/AST >8 times the ULN

3. Development of a clinical condition requiring immediate treatment with a statin

4. Discharge from critical care environment

5. Death

6. Discontinuation of active medical treatment

7. Patient or representative request for withdrawal of patient from the study

8. Decision by the attending clinician that the study drug should be discontinued on safety grounds

### Study drug compliance

Any omission of the study drug will be recorded in the Case Report Form (CRF) to monitor treatment compliance.

### Clinical management of patients in the trial

Patients involved in the trial will be managed according to best practice established locally on each unit. Clinicians will be encouraged to use a low tidal volume strategy of ventilation based on ideal body weight, a conservative fluid management protocol and a standardized weaning strategy. Rescue therapies such as high frequency oscillatory ventilation, nitric oxide and extracorporeal membrane oxygenation can be used according to local policy.

The exclusion criteria prevent patients with ALI who have a co-existing condition that requires treatment with a statin as part of standard clinical care being recruited. In patients where there is a clinical indication for acute and immediate treatment with a statin after randomization, for example, acute myocardial infarction, the study drug will be discontinued and a statin commenced. The patient will not be unblinded and data collection will continue. This will be recorded on the CRF. Otherwise, patients will not be commenced on a statin for the duration of the clinical trial.

### Study procedures for unblinding

As a placebo controlled, double-blind trial, patients, clinicians and research staff will be blinded to which arm of the study a patient is allocated. All trial drugs will be packaged identically and identified only by a unique trial identifier. The protocol allows for emergency unblinding in the event of significant concerns about patient safety. This option will be used if the patient’s future treatment requires knowledge of the treatment assignment. If a local investigator decides that there is justification to unblind a patient, they should make every attempt to contact the CTU, who will arrange for them to discuss unblinding with a clinical member of the trial team. Emergency unblinding will be performed by telephone contact with the randomization service. All events will be logged.

### Data collection

All data for an individual patient will be recorded in the study CRF. The majority of data will be obtained from the patient’s hospital record. Data will be collected by the site research team until hospital discharge. In the event that a patient is transferred to another hospital, the site research team will liaise with the receiving hospital to ensure complete data collection. The EQ-5D will be administered face-to-face at discharge. The EQ-5D and resource use questionnaires will then be administered by post or by telephone after the patient has been discharged from hospital. On discharge the participating site will provide the trial manager at the CTU with the name, address and contact details for the patient. Postal health-related quality of life and resource utilization questionnaires will be sent out and collected by the CTU. If questionnaires are not returned telephone contact will be made to the trial patient to check that the questionnaire has been received and the patient is happy to complete it, followed by a second copy of the questionnaire. If the second questionnaire is not returned the patient will be contacted by telephone and the outcome data collected over the telephone. Trial patients will be asked to let the CTU know if they move house at any time after hospital discharge.

Patient identification on the CRF and questionnaires will be through their unique trial identifier allocated at the time of randomization and patient initials.

### Monitoring and reporting adverse events

HARP-2 is recruiting a population that is already in a life-threatening situation; it is expected that many of the participants will experience adverse events (AEs). Events that are expected in this population (that is, events that are in keeping with the patient’s underlying medical condition) should not be reported as AEs. If any AEs are related to the study drug (that is, are adverse reactions) they must be reported on the AE form within the CRF.

SAEs thought to be related to the study drug or SUSARs (that is, their nature or severity is not consistent with the summary of product characteristics for simvastatin) will be reported to the CTU within 24 hours of becoming aware of their occurrence. The CTU will inform the sponsor and regulatory authorities within the required timelines as per the regulatory requirements.

### End of trial

The trial will end when 540 patients have been recruited. The trial will be stopped prematurely if: mandated by the Research Ethics Committee, the Medicines and Healthcare products Regulatory Agency (MHRA), the Irish Medicines Board (IMB), the Sponsor (for example, following recommendations from the Data Monitoring and Ethics Committee (DMEC) or funding for the trial ceases.

### Statistical analysis plan

Standard approaches will be used to detect patterns in missing data. Analyses will be on an intention-to-treat basis. As VFDs are unlikely to be normally distributed, the groups will be analyzed by comparing the medians and 95% confidence intervals (CI). The comparison of other continuous outcomes will be by analysis of variance, including covariates where appropriate. Statistical diagnostic methods will be used to check for violations of the assumptions, and transformations will be performed where required. A statistical interaction test will be used to assess differences in treatment effects between the subgroups. For binary outcome measures risk ratios and associated 95% CI will be calculated. Binary variables assessed daily will be analyzed using logistic regression analysis corrected for days at risk. Time-to-event outcomes will be analyzed by survival methods and reported as hazard ratios with 95% CI. An interim analysis of efficacy is not planned.

### Subgroup analyses

Four subgroup analyses are planned to analyze whether treatment effect is modified by age, vasopressor requirement (defined as any inotropic requirement except dopamine <6mcg/kg/minute); etiology of ALI (due to sepsis versus non-sepsis) and CRP level at baseline. Subgroup analyses will use a statistical test for interaction and will be reported using 99% CI. The trial statistician has written a detailed statistical analysis plan (SAP).

### Health economic evaluation

A within-trial cost effectiveness analysis (CEA) will be undertaken to compare the costs and outcomes of patients in each arm of the trial at 12 months follow-up (post-randomization). A health service perspective will be adopted for this analysis as recommended by the National Institute for Health and Clinical Excellence (NICE) [22] with additional information being collected relating to social care costs. The outcome for the analysis will be the Quality Adjusted Life Year (QALY) and utilities will be measured using the EQ-5D at discharge, 3, 6 and 12 months. Resource utilization will be collected at 6 and 12 months only. Administration of the EQ-5D (at four separate time points) has been undertaken to ensure that any utility differences between arms will be fully captured.

Consistent with the perspective chosen for the analysis, resource utilization will be quantified (at all sites to allow evaluation of cost-effectiveness in both jurisdictions); however, the focus of the proposed evaluation will be to determine cost-effectiveness within a UK context. Hence unit costs will be applied from national sources such as the National Health Service (NHS) reference costs, British National Formulary (BNF) and the Personal Social Services Research Unit (PSSRU) Unit Costs of Health and Social Care [23]. Where national costs are not available, unit costs will be identified in consultation with finance departments of hospitals/Trusts. Patient-specific resource utilization (of primary, community and social care services) will be extracted from the trial CRF and via self-completed patient questionnaires. It will not be necessary to discount costs and outcomes (for the within-trial analysis) given the duration of follow-up. Parameter uncertainty will be addressed using sensitivity analysis. Outputs from the analysis will include the incremental cost effectiveness ratio (ICER), a scatter plot on the cost effectiveness plane, cost effectiveness acceptability curve (CEAC) and incremental net benefit (INB) assuming a societal willingness-to-pay of £20,000/QALY or the Republic of Ireland (ROI) equivalent.

### Trial oversight

The Chief Investigators will have overall responsibility for the conduct of the study. The Trial Management Group will have responsibility for the day to day operational management of the trial. Trial oversight will be provided by a Trial Steering Committee (TSC) comprising investigators, clinicians and trialists. The TSC will operate within the relevant CTU Standard Operating Procedure (SOP). An independent Data Monitoring and Ethics Committee (DMEC) will monitor the safety of participants enrolled in the trial through regular review of adverse event reports. The reports provided to the DMEC will include information on the AEs reported, deaths from all causes at 28 days and recruitment, along with any other data that the committee may request. The DMEC will advise the TSC if, in their view, the randomized comparisons have provided both (i) 'proof beyond reasonable doubt' that for all, or some, the treatment is clearly indicated or clearly contra-indicated and (ii) evidence that might reasonably be expected to materially influence future patient management. Following a report from the DMEC, the TSC will decide what actions, if any, are required. Unless the DMEC requests cessation of the trial the TSC and the collaborators will not be informed of the reports provided to the DMEC.

## Trial status

As of December 2012, 293 patients are currently enrolled from 34 ICUs. Recruitment will continue to May 2014.

## Abbreviations

AE: adverse event; ALI: acute lung injury; ALT: alanine transaminase; AST: aspartate aminotransferase; BHSCT: Belfast Health and Social Care Trust; BNF: British National Formulary; CEAC: cost effectiveness acceptability curve; CI: confidence interval; CONSORT: Consolidated Standards of Reporting Trials; CRF: case report form; CTU: Clinical Trials Unit; DMEC: Data Monitoring and Ethics Committee; EQ-5D: EuroQol 5 dimension questionnaire; GCP: good clinical practice; HRQOL: Health Related Quality of Life; ICER: incremental cost-effectiveness ratio; ICNARC: Intensive Care National Audit and Research Centre; IL: interleukin; IMB: Irish Medicines Board; INB: incremental net benefit; ISRCTN: International Standardised Randomised Controlled Trial Number; MHRA: Medicines and Healthcare products Regulatory Agency; MRC: Medical Research Council; NFκB: nuclear factor kappa B; NHS: National Health Service; NICE: National Institute for Health and Clinical Excellence; NIHR: National Institute for Health Research; PSSRU: Personal Social Service Research Unit; QALY: quality adjusted life years; ROI: Republic of Ireland; SAE: serious adverse event; SOP: standard operating procedure; SUSAR: suspected unexpected serious adverse reaction; ULN: upper limit of normal; VFDs: ventilator free days.

## Competing interests

DM has consulted for, sat on advisory boards for, and received lecture fees from GlaxoSmithKline, and received lecture fees from AstraZeneca for educational meetings (all unrelated to statins). All other authors declare that they have no competing interests.

## Authors’ contributions

DM conceived the study. All authors made a substantial contribution to the protocol development. All authors read and approved the final manuscript.
